# Colloid Adenocarcinoma of the Lung: A Rare and Unique Entity

**DOI:** 10.7759/cureus.82586

**Published:** 2025-04-19

**Authors:** Uma Bhatta, Leslie Lopez-Calderon

**Affiliations:** 1 Pathology and Laboratory Medicine, University of Tennessee Health Science Center, Memphis, USA

**Keywords:** colloid adenocarcinoma, immunohistochemistry, lung adenocarcinoma, mucinous adenocarcinoma, surgical resection

## Abstract

Primary pulmonary colloid adenocarcinoma (PPCA) is a rare variant of lung adenocarcinoma characterized by abundant extracellular mucin production, often posing diagnostic challenges due to its slow growth and nonspecific imaging findings. We present the case of a 77-year-old male with a medical history of coronary artery disease (CAD) and congestive heart failure (CHF), who exhibited a gradually enlarging right lower lobe lung mass over eight years. Initial imaging and biopsy results were inconclusive, but a subsequent biopsy following surgical resection confirmed the diagnosis of PPCA. This case highlights the clinical challenges, pathological features, and diagnostic complexities for elderly patients with this rare type of lung carcinoma. It emphasizes the crucial role of surgical resection in its management.

## Introduction

Primary pulmonary colloid adenocarcinoma (PPCA) represents an exceedingly rare histological subtype of lung adenocarcinoma, accounting for approximately 0.24% of all primary lung malignancies [1-4]. This distinct entity is characterized by scant neoplastic epithelium and extensive extracellular mucin accumulation in pools within the alveolar spaces, which distend alveolar walls and destroy them [5,6]. The indolent clinical presentation coupled with nonspecific radiographic features frequently necessitates comprehensive histopathological and immunohistochemical evaluation for accurate diagnosis [4]. Despite an indolent clinical course, tumor recurrence can occur even in early-stage cases.

Immunohistochemically, PPCA typically demonstrates tumor cells positive for CK7, with variable expression of CK20. These biomarkers aid in confirming its primary pulmonary origin [1,2]. Given its morphological similarities to metastatic mucinous adenocarcinomas from other sites, such as the gastrointestinal tract, accurate differentiation is important [3]. Surgical resection is the primary treatment modality, providing definitive diagnostic confirmation and therapeutic intervention [7]. We report a case of PPCA in a 77-year-old male with multiple comorbidities to illustrate the diagnostic challenges and management strategies associated with this rare malignancy.

## Case presentation

A 77-year-old male with a past medical history significant for coronary artery disease with congestive heart failure was monitored for an enlarging mass in the right lower lung lobe, initially observed in 2016. Computed tomography (CT) showed the lesion progressed from 11 × 10 × 14 mm to 27 × 16 mm by 2020. A subsequent fluorodeoxyglucose positron emission tomography (FDG-PET) scan in 2021 revealed a non-FDG avid nodule surrounded by a halo of consolidation. There was no evidence of regional lymphadenopathy or distant metastasis, and a CT-guided biopsy proved non-diagnostic. The patient initially declined surgical intervention.

By June 2024, a follow-up CT scan showed the mass had grown to 28 × 17 mm, appearing as an irregular subpleural mass in the medial right lower lobe. A subsequent CT-guided biopsy revealed abundant mucin pools with few atypical cells. The cells appeared well-differentiated, with minimal nuclear pleomorphism. Despite the increasing size of the lesion, there was no evidence of suspicious mediastinal, hilar, or axillary lymphadenopathy.

**Figure 1:**
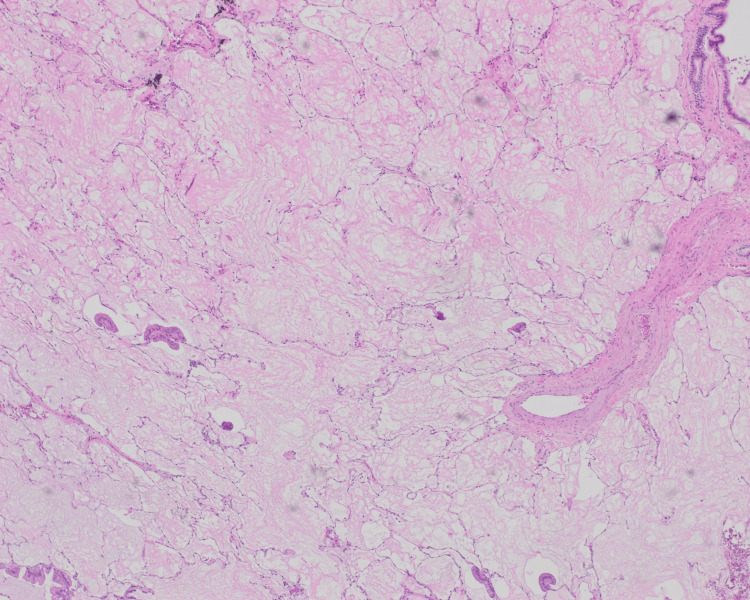
Colloid adenocarcinoma shows abundant pools of mucin distending alveolar septa with scant floating single cells and small islands of neoplastic cells in between residual alveolar walls (H&E, 4×).

**Figure 2:**
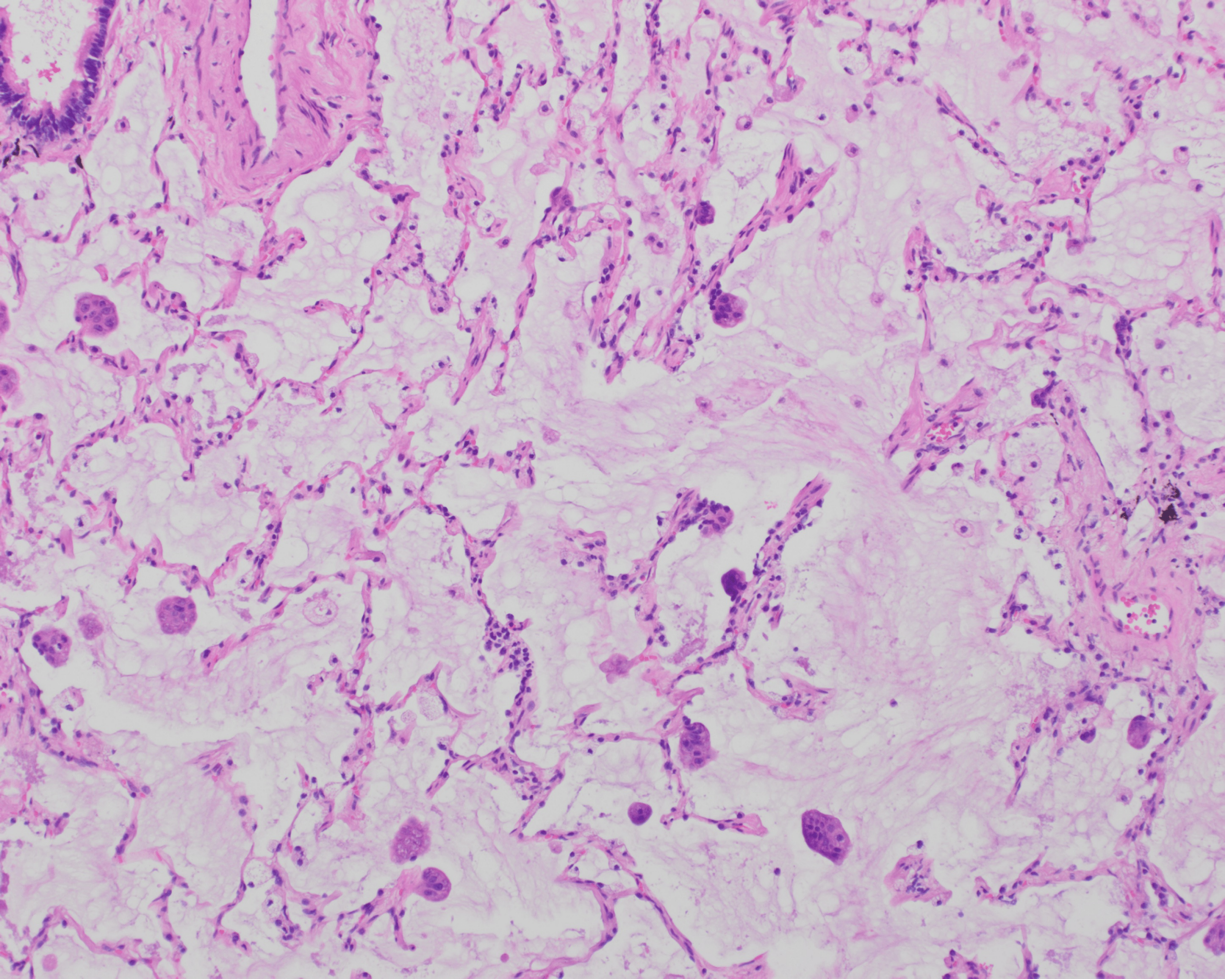
Entrapped alveolar septa can be seen, distended and destroyed by large amounts of mucinous material. Small clusters of well-differentiated tumor cells are floating within the pools of mucin (H&E, 10×).

**Figure 3:**
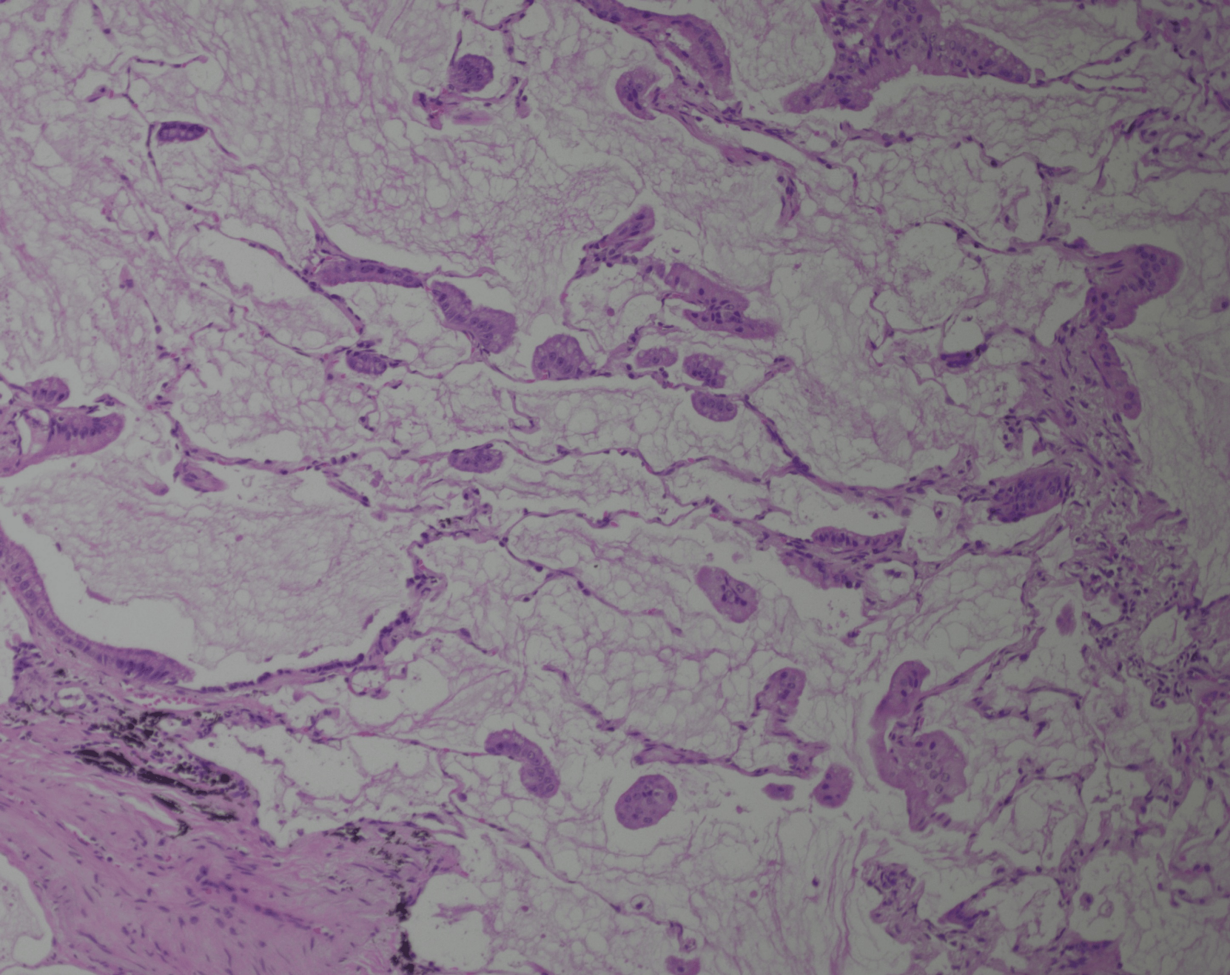
Colloid adenocarcinoma comprised clusters of mucinous glandular epithelium floating in the mucin pools and growing along the surface of alveolar septa. Note that the tumor cells do not completely line the alveolar spaces (H&E, 10×).

**Figure 4:**
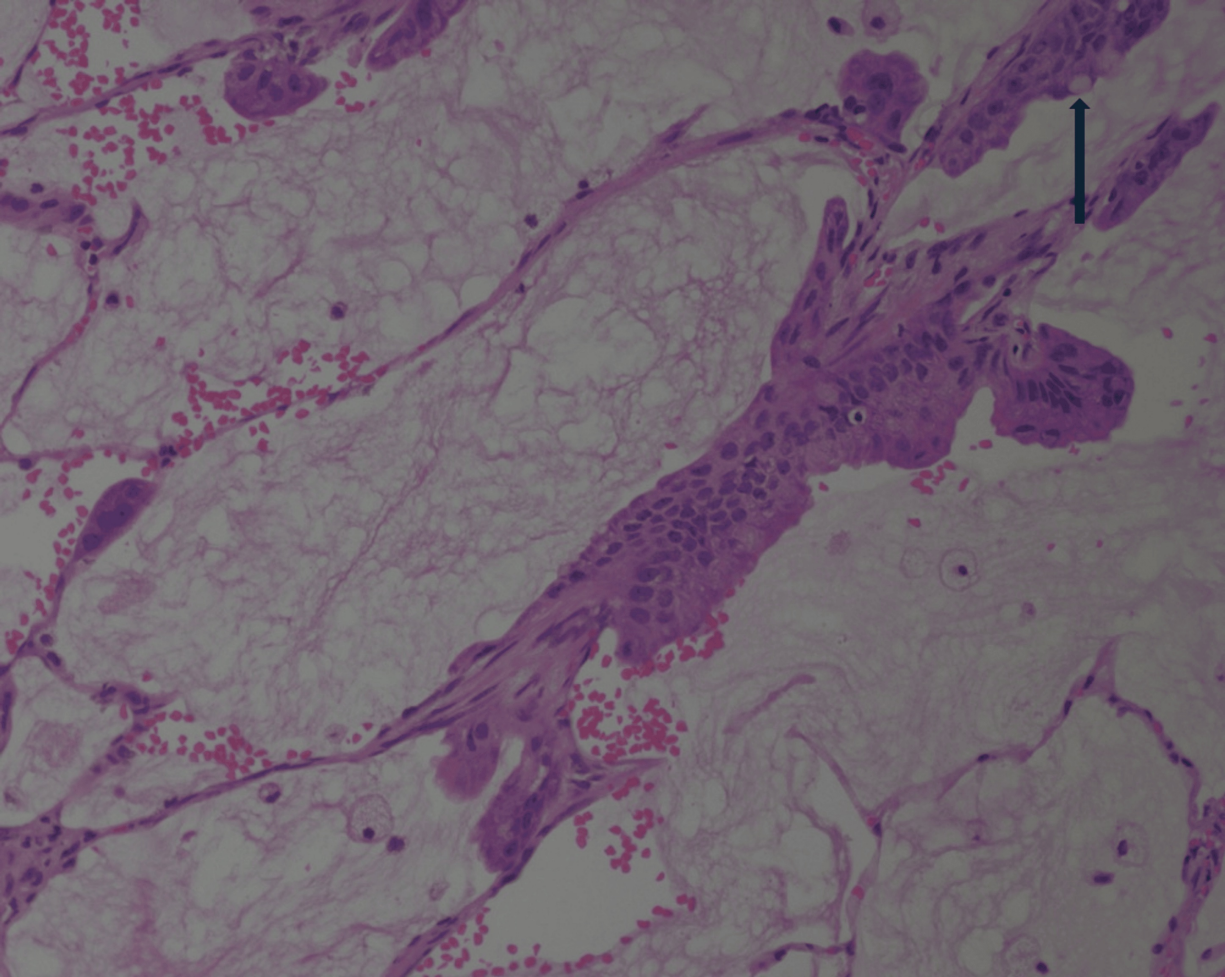
Colloid adenocarcinoma comprised columnar neoplastic cells with focal goblet cell formation (shown by arrow) lining and growing along alveolar wall (H&E, 20×).

**Figure 5:**
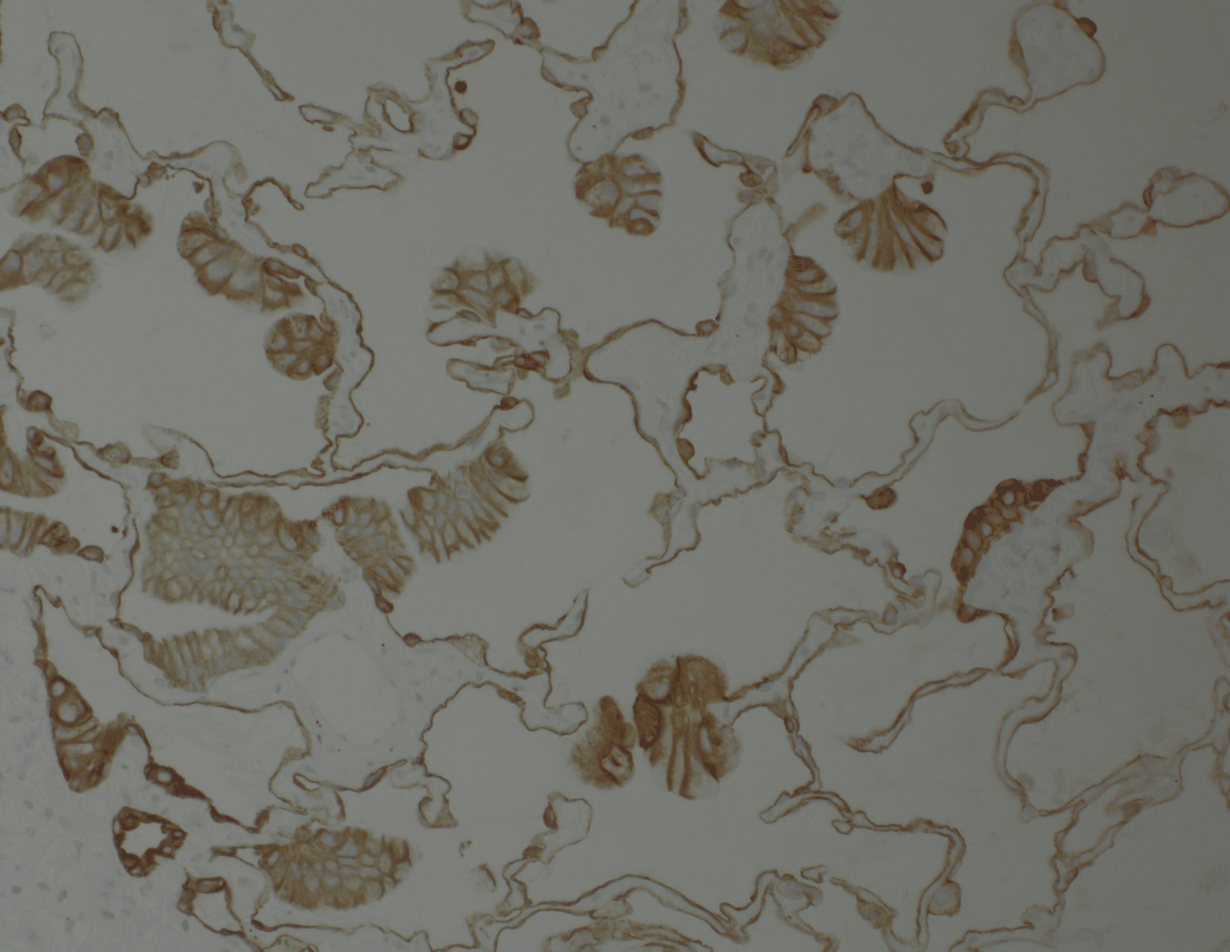
Immunohistochemistry (IHC) demonstrated tumor cells strongly and diffusely positive for CK7 (20×).

In November 2024, the patient underwent a right thoracotomy with wedge resection. Macroscopic examination revealed a soft, well-circumscribed tumor mass measuring 35 × 30 × 25 mm close to the pleural surface with a glistening, tan-white, gelatinous cut surface with cystic spaces. Microscopic examination confirmed the diagnosis of colloid adenocarcinoma with abundant pools of mucin with floating single cells and small islands of neoplastic cells in between residual alveolar septa, and extracellular mucin pools destroying the alveolar spaces (Figures 1-3). Tumor cells grow as columnar, well-differentiated epithelium with goblet cell changes (Figure 4). Immunohistochemistry demonstrated tumor cells strongly and diffusely positive for CK7 (Figure 5), patchy and weakly positive for CK20, and negative for TTF-1. The patient experienced an uneventful postoperative recovery and was subsequently discharged.

## Discussion

PPCA is an uncommon subtype of lung adenocarcinoma characterized by abundant extracellular mucin production, destroying alveolar spaces [1-4]. PPCA commonly occurs in individuals aged 35 to 86 years, aligning with the age range of our case. The average tumor size is reported to be 3.0 cm, with a range of 1.0 to 8.2 cm [1]. The diagnosis is often complicated by slow growth and nonspecific imaging, requiring histopathological confirmation after resection [2,4]. In our case, the patient's mass exhibited non-FDG avidity on the PET scan, and initial biopsies yielded inconclusive results, underscoring the inherent diagnostic challenges associated with PPCA. Immunohistochemistry is of little utility in colloid adenocarcinoma because of the overlapping staining between mucinous neoplasms arising in the lung and GI tract [3,5]. Typically, PPCA demonstrates tumor cells positive for CK7, CK20, and CDX-2 [5,6]. In this particular case, the absence of a gastrointestinal malignancy and the presence of abundant extracellular mucin pools distorting alveolar architecture, combined with strong CK7 immunoreactivity, support the diagnosis of PPCA.

Surgical resection is the primary treatment modality for PPCA, offering diagnostic confirmation and therapeutic advantages [1,4]. In this patient, wedge resection from the right lower lobe of the lung not only provided a definitive diagnosis but also ensured complete tumor removal with clear margins.

The rarity of PPCA means that it is not always included in the differential diagnosis of lung cancer. This is especially true for elderly patients, where the clinical presentation may overlap with more common conditions such as chronic obstructive pulmonary disease (COPD) or cardiovascular disease. In this case, the patient's underlying cardiovascular disease further complicated the clinical presentation of the lung mass.

This report underscores the diagnostic complexities and the significance of surgical resection and immunohistochemistry in differentiating PPCA from other mucinous adenocarcinomas. The World Health Organization (WHO) Classification of Thoracic Tumors (2021) provides a valuable framework for subtyping rare lung tumors, including PPCA [5]. Although PPCA rarely recurs or metastasizes, it generally carries a favorable prognosis with complete surgical resection [3,7].

## Conclusions

Although colloid adenocarcinoma is generally considered to have an indolent clinical behavior, tumor recurrence can occur even in early-stage cases. The treatment strategy for colloid adenocarcinoma should follow the guidelines for primary lung cancer.
